# Carb­oxy­methyl ursolate monohydrate

**DOI:** 10.1107/S1600536811001619

**Published:** 2011-01-15

**Authors:** Yan Zhong, ZhaoHui Dai, YanBo Teng, Bin Wu, JianPing Zhou

**Affiliations:** a School of Pharmacy, China Pharmaceutical University, Tongjiaxiang No. 24 Nanjing, Nanjing 210009, People’s Republic of China; b School of Chemistry and Chemical Engineering, Southeast University, Sipailou No. 2 Nanjing, Nanjing 210096, People’s Republic of China; c School of Pharmacy, Nanjing Medical University, Hanzhong Road No.140 Nanjing, Nanjing 210029, People’s Republic of China

## Abstract

In the title compound, C_28_H_50_O_5_·H_2_O, all of the six-membered rings of the penta­cyclic triterpene skeleton adopt chair conformations. In the crystal, mol­ecules are linked by O—H⋯O and C—H⋯O hydrogen bonds.

## Related literature

For the synthesis, see: Wen *et al.* (2006 ▶). The crystal structure of ursolic acid is known from its ethanol solvate, see: Simon *et al.* (1992 ▶). For methyl uroslate-3-bromo­acetate, see: Stout & Stevens (1963 ▶). For methyl ursolate-3-*p*-bromo­benzoate, see: Paton & Paul (1979 ▶). For background to ursolic acid derivatives and their biological activity, see: Es-saady *et al.* (1996 ▶). For bond-length data, see: Allen *et al.* (1987 ▶).
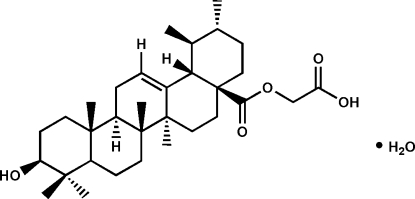


## Experimental

### 

#### Crystal data

C_32_H_50_O_5_·H_2_O
*M* *_r_* = 532.74
Monoclinic, 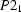

*a* = 13.338 (3) Å
*b* = 8.1010 (16) Å
*c* = 14.311 (3) Å
β = 106.26 (3)°
*V* = 1484.5 (5) Å^3^
*Z* = 2
Mo *K*α radiation
μ = 0.08 mm^−1^
*T* = 293 K
0.30 × 0.20 × 0.10 mm

#### Data collection

Enraf–Nonius CAD-4 diffractometer
Absorption correction: ψ scan (North *et al.*, 1968 ▶) *T*_min_ = 0.976, *T*_max_ = 0.992
5692 measured reflections
5446 independent reflections
4536 reflections with *I* > 2σ(*I*)
*R*_int_ = 0.025
3 standard reflections every 200 reflections  intensity decay: 1%

#### Refinement

*R*[*F* ^2^ > 2σ(*F* ^2^)] = 0.048
*wR*(*F* ^2^) = 0.134
*S* = 1.00
5446 reflections
350 parameters
1 restraint
H-atom parameters constrained
Δρ_max_ = 0.18 e Å^−3^
Δρ_min_ = −0.23 e Å^−3^

### 

Data collection: *CAD-4 EXPRESS* (Enraf–Nonius, 1994) ▶; cell refinement: *CAD-4 EXPRESS*; data reduction: *XCAD4* (Harms & Wocadlo, 1995 ▶); program(s) used to solve structure: *SHELXS97* (Sheldrick, 2008 ▶); program(s) used to refine structure: *SHELXL97* (Sheldrick, 2008 ▶); molecular graphics: *SHELXTL* (Sheldrick, 2008 ▶); software used to prepare material for publication: *SHELXTL*.

## Supplementary Material

Crystal structure: contains datablocks I, global. DOI: 10.1107/S1600536811001619/hb5787sup1.cif

Structure factors: contains datablocks I. DOI: 10.1107/S1600536811001619/hb5787Isup2.hkl

Additional supplementary materials:  crystallographic information; 3D view; checkCIF report

## Figures and Tables

**Table 1:** Hydrogen-bond geometry (Å, °)

*D*—H⋯*A*	*D*—H	H⋯*A*	*D*⋯*A*	*D*—H⋯*A*
O*W*—H*WB*⋯O2^i^	0.85	2.41	2.839 (3)	112
O*W*—H*WB*⋯O4^i^	0.85	2.58	3.365 (3)	154
O1—H1*A*⋯O4^ii^	0.82	1.98	2.758 (3)	159
O*W*—H*WA*⋯O1^iii^	0.86	1.88	2.681 (3)	154
O5—H5*B*⋯O*W*	0.85	1.74	2.575 (3)	165
C12—H12*A*⋯O*W*^i^	0.93	2.57	3.455 (3)	159

Symmetry codes: (i) 
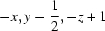
; (ii) 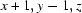; (iii) 
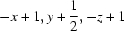
.

**Fig. 1.**
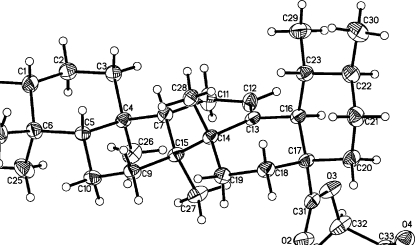
The molecular structure of (I), showing displacement ellipsoids at the 70% probability level.

**Fig. 2.**
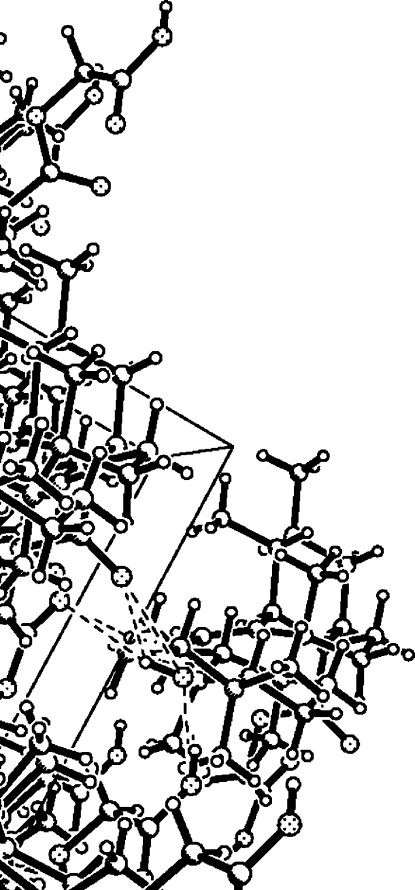
A packing diagram of (I).

## supplementary crystallographic information

### Experimental

To a solution of ursolic acid (5.0 g, 11 mmol) in DMF (125 ml) were added ethyl chloroacetate (1.17 ml, 11 mmol) and K2CO3 (4.5 g, 33 mmol). The reaction mixture was stirred at room temperature for 5 h, then filtered and concentrated to give ethoxycarbonylmethyl ursolate as a yellow oil, which is pure enough for the next step. The ester was dissolved in MeOH (50 ml), THF (75 ml) and 4 N NaOH (30 ml). The mixture was stirred at room temperature for 18 h. After solvent evaporation under reduced pressure, H2O (90 ml) was added. The mixture was acidified with 4 N HCl until the pH was adjusted to 3.0 and the product was extracted with CHCl_3_ (75 ml). The organic layer was separated and dried in Na_2_SO_4_. Filtration and concentration under reduce pressure provided carboxymethyl ursolate as a white powder. The colorless block of the title compound used in *x*-ray diffraction studies were grown in a mixture of ethyl acetate and hexane by a slow evaporation at room temperature.

### Refinement

The absolute sturucture was indeterminate in the present experiment. The relative chiralities of the stereogenic centres are: C1 R*, C4 S*, C5 S*, C7 R*, C14 R*, C15 S*, C16 R*, C17 R*, C22 S*, C23 R*. All H atoms bonded to the C atoms were placed geometrically at the distances of 0.93–0.97 Å, and included in the refinement in riding motion approximation with *U*ĩso~(H) = 1.2 or 1.5*U*~eq~ of the carrier atom.

### Crystal data

**Table:**

C_32_H_50_O_5_·H_2_O	*F*(000) = 584
*M**_r_* = 532.74	*D*_x_ = 1.192 Mg m^−^^3^
Monoclinic, *P*2_1_	Mo *K*α radiation, λ = 0.71073 Å
Hall symbol: P 2yb	Cell parameters from 25 reflections
*a* = 13.338 (3) Å	θ = 9–13°
*b* = 8.1010 (16) Å	µ = 0.08 mm^−^^1^
*c* = 14.311 (3) Å	*T* = 293 K
β = 106.26 (3)°	Block, colorless
*V* = 1484.5 (5) Å^3^	0.30 × 0.20 × 0.10 mm
*Z* = 2	

### Data collection

**Table:**

Enraf–Nonius CAD-4 diffractometer	4536 reflections with *I* > 2σ(*I*)
Radiation source: fine-focus sealed tube	*R*_int_ = 0.025
graphite	θ_max_ = 25.4°, θ_min_ = 1.5°
ω/2θ scans	*h* = 0→16
Absorption correction: ψ scan (North *et al.*, 1968)	*k* = −9→9
*T*_min_ = 0.976, *T*_max_ = 0.992	*l* = −17→16
5692 measured reflections	3 standard reflections every 200 reflections
5446 independent reflections	intensity decay: 1%

### Refinement

**Table:**

Refinement on *F*^2^	Primary atom site location: structure-invariant direct methods
Least-squares matrix: full	Secondary atom site location: difference Fourier map
*R*[*F*^2^ > 2σ(*F*^2^)] = 0.048	Hydrogen site location: inferred from neighbouring sites
*wR*(*F*^2^) = 0.134	H-atom parameters constrained
*S* = 1.00	*w* = 1/[σ^2^(*F*_o_^2^) + (0.094*P*)^2^] where *P* = (*F*_o_^2^ + 2*F*_c_^2^)/3
5446 reflections	(Δ/σ)_max_ < 0.001
350 parameters	Δρ_max_ = 0.18 e Å^−^^3^
1 restraint	Δρ_min_ = −0.23 e Å^−^^3^

### Special details

**Table:**

Geometry. All e.s.d.'s (except the e.s.d. in the dihedral angle between two l.s. planes) are estimated using the full covariance matrix. The cell e.s.d.'s are taken into account individually in the estimation of e.s.d.'s in distances, angles and torsion angles; correlations between e.s.d.'s in cell parameters are only used when they are defined by crystal symmetry. An approximate (isotropic) treatment of cell e.s.d.'s is used for estimating e.s.d.'s involving l.s. planes.
Refinement. Refinement of *F*^2^ against ALL reflections. The weighted *R*-factor *wR* and goodness of fit *S* are based on *F*^2^, conventional *R*-factors *R* are based on *F*, with *F* set to zero for negative *F*^2^. The threshold expression of *F*^2^ > σ(*F*^2^) is used only for calculating *R*-factors(gt) *etc*. and is not relevant to the choice of reflections for refinement. *R*-factors based on *F*^2^ are statistically about twice as large as those based on *F*, and *R*- factors based on ALL data will be even larger.

### Fractional atomic coordinates and isotropic or equivalent isotropic displacement parameters (Å^2^)

**Table:**

	*x*	*y*	*z*	*U*_iso_*/*U*_eq_	
O1	0.85435 (16)	0.1230 (3)	0.65587 (19)	0.0785 (7)	
H1A	0.8627	0.0229	0.6621	0.118*	
C1	0.7706 (2)	0.1725 (3)	0.6936 (2)	0.0484 (6)	
H1B	0.7911	0.1490	0.7636	0.058*	
C2	0.6748 (2)	0.0714 (3)	0.64682 (19)	0.0456 (6)	
H2A	0.6903	−0.0447	0.6601	0.055*	
H2B	0.6557	0.0871	0.5769	0.055*	
O2	0.16556 (16)	0.8730 (2)	0.72389 (16)	0.0640 (6)	
O3	0.09919 (14)	0.6206 (2)	0.68621 (12)	0.0547 (5)	
C3	0.58375 (18)	0.1207 (3)	0.68505 (18)	0.0401 (5)	
H3A	0.6009	0.0951	0.7539	0.048*	
H3B	0.5231	0.0557	0.6521	0.048*	
C4	0.55611 (17)	0.3049 (3)	0.67043 (15)	0.0359 (5)	
O4	−0.07654 (15)	0.8025 (2)	0.65381 (14)	0.0564 (5)	
C5	0.65734 (17)	0.4056 (3)	0.71361 (16)	0.0343 (5)	
H5A	0.6771	0.3794	0.7832	0.041*	
O5	−0.10926 (14)	0.7942 (3)	0.49516 (14)	0.0579 (5)	
H5B	−0.0879	0.7665	0.4467	0.069*	
C6	0.75574 (19)	0.3587 (3)	0.68039 (18)	0.0425 (6)	
C7	0.48023 (17)	0.3472 (3)	0.73329 (16)	0.0343 (5)	
H7A	0.5160	0.3067	0.7987	0.041*	
C9	0.56111 (19)	0.6301 (3)	0.77258 (17)	0.0404 (5)	
H9A	0.5449	0.7470	0.7669	0.048*	
H9B	0.5987	0.6085	0.8399	0.048*	
C10	0.6324 (2)	0.5904 (3)	0.70921 (18)	0.0424 (6)	
H10A	0.6968	0.6529	0.7316	0.051*	
H10B	0.5987	0.6221	0.6425	0.051*	
C11	0.3777 (2)	0.2509 (3)	0.7033 (2)	0.0544 (7)	
H11A	0.3494	0.2572	0.6332	0.065*	
H11B	0.3920	0.1357	0.7204	0.065*	
C12	0.29722 (17)	0.3121 (3)	0.74972 (17)	0.0420 (5)	
H12A	0.2350	0.2531	0.7364	0.050*	
C13	0.30539 (16)	0.4407 (3)	0.80729 (15)	0.0330 (5)	
C14	0.40501 (17)	0.5457 (3)	0.83295 (15)	0.0326 (5)	
C15	0.45858 (18)	0.5331 (3)	0.74771 (16)	0.0353 (5)	
C16	0.21627 (17)	0.4787 (3)	0.85160 (16)	0.0350 (5)	
H16A	0.1534	0.4292	0.8080	0.042*	
C17	0.19584 (18)	0.6672 (3)	0.85059 (17)	0.0373 (5)	
C18	0.29810 (19)	0.7566 (3)	0.90141 (18)	0.0416 (5)	
H18A	0.2845	0.8739	0.9037	0.050*	
H18B	0.3234	0.7172	0.9679	0.050*	
C19	0.38151 (19)	0.7295 (3)	0.84990 (17)	0.0390 (5)	
H19A	0.4456	0.7814	0.8878	0.047*	
H19B	0.3602	0.7847	0.7873	0.047*	
C20	0.1086 (2)	0.7089 (3)	0.89839 (19)	0.0486 (6)	
H20A	0.1045	0.8278	0.9044	0.058*	
H20B	0.0423	0.6708	0.8564	0.058*	
C21	0.1263 (2)	0.6317 (4)	0.99763 (19)	0.0533 (7)	
H21A	0.0679	0.6580	1.0229	0.064*	
H21B	0.1889	0.6780	1.0417	0.064*	
C22	0.1377 (2)	0.4456 (3)	0.99400 (18)	0.0480 (6)	
H22A	0.0736	0.4010	0.9498	0.058*	
C23	0.22925 (19)	0.4004 (3)	0.95339 (17)	0.0411 (5)	
H23A	0.2937	0.4431	0.9981	0.049*	
C24	0.8518 (2)	0.4416 (4)	0.7495 (2)	0.0584 (7)	
H24A	0.8526	0.4201	0.8157	0.088*	
H24B	0.8486	0.5585	0.7381	0.088*	
H24C	0.9141	0.3979	0.7378	0.088*	
C25	0.7499 (3)	0.4133 (4)	0.5764 (2)	0.0605 (7)	
H25A	0.8161	0.3942	0.5640	0.091*	
H25B	0.7333	0.5288	0.5692	0.091*	
H25C	0.6967	0.3512	0.5310	0.091*	
C26	0.5071 (2)	0.3335 (4)	0.56091 (17)	0.0514 (7)	
H26A	0.5437	0.2691	0.5247	0.077*	
H26B	0.5120	0.4484	0.5463	0.077*	
H26C	0.4350	0.3010	0.5434	0.077*	
C27	0.3843 (2)	0.6049 (4)	0.65303 (17)	0.0512 (6)	
H27A	0.4132	0.5853	0.5996	0.077*	
H27B	0.3765	0.7215	0.6606	0.077*	
H27C	0.3173	0.5522	0.6402	0.077*	
C28	0.47549 (17)	0.4806 (3)	0.93108 (16)	0.0402 (5)	
H28A	0.4440	0.5058	0.9821	0.060*	
H28B	0.5428	0.5323	0.9449	0.060*	
H28C	0.4833	0.3632	0.9272	0.060*	
C29	0.2394 (3)	0.2128 (4)	0.9468 (2)	0.0610 (8)	
H29A	0.2534	0.1656	1.0107	0.092*	
H29B	0.2957	0.1871	0.9197	0.092*	
H29C	0.1755	0.1681	0.9059	0.092*	
C30	0.1508 (3)	0.3706 (4)	1.0951 (2)	0.0671 (8)	
H30A	0.0986	0.4151	1.1225	0.101*	
H30B	0.2189	0.3967	1.1365	0.101*	
H30C	0.1429	0.2529	1.0894	0.101*	
C31	0.15463 (19)	0.7326 (3)	0.74804 (19)	0.0441 (6)	
C32	0.0496 (2)	0.6730 (4)	0.58938 (19)	0.0587 (8)	
H32A	0.0359	0.5778	0.5466	0.070*	
H32B	0.0960	0.7464	0.5676	0.070*	
C33	−0.0518 (2)	0.7612 (3)	0.58279 (19)	0.0435 (6)	
OW	−0.04995 (14)	0.6584 (3)	0.35682 (15)	0.0641 (6)	
HWB	−0.0315	0.5578	0.3656	0.077*	
HWA	0.0065	0.6702	0.3393	0.077*	

### Atomic displacement parameters (Å^2^)

**Table:**

	*U*^11^	*U*^22^	*U*^33^	*U*^12^	*U*^13^	*U*^23^
O1	0.0566 (12)	0.0473 (12)	0.149 (2)	0.0031 (10)	0.0575 (14)	−0.0128 (13)
C1	0.0439 (14)	0.0373 (13)	0.0673 (16)	0.0051 (11)	0.0211 (13)	−0.0026 (12)
C2	0.0506 (15)	0.0300 (12)	0.0590 (15)	0.0019 (11)	0.0198 (12)	−0.0073 (11)
O2	0.0589 (12)	0.0472 (11)	0.0797 (14)	0.0059 (9)	0.0092 (10)	0.0249 (10)
O3	0.0641 (12)	0.0464 (10)	0.0434 (9)	0.0193 (9)	−0.0018 (8)	0.0006 (8)
C3	0.0411 (12)	0.0271 (11)	0.0542 (13)	−0.0019 (10)	0.0168 (11)	−0.0084 (10)
C4	0.0370 (11)	0.0288 (11)	0.0405 (12)	0.0031 (10)	0.0084 (9)	−0.0033 (10)
O4	0.0618 (12)	0.0465 (10)	0.0634 (12)	0.0084 (10)	0.0216 (9)	−0.0023 (9)
C5	0.0372 (12)	0.0294 (11)	0.0362 (11)	−0.0002 (9)	0.0101 (9)	0.0005 (9)
O5	0.0508 (10)	0.0576 (12)	0.0575 (11)	0.0168 (10)	0.0024 (8)	0.0007 (9)
C6	0.0440 (13)	0.0324 (12)	0.0562 (15)	−0.0022 (10)	0.0222 (12)	−0.0042 (11)
C7	0.0353 (12)	0.0282 (12)	0.0380 (11)	−0.0007 (9)	0.0077 (9)	−0.0044 (9)
C9	0.0486 (13)	0.0246 (11)	0.0507 (13)	−0.0019 (10)	0.0183 (11)	−0.0061 (10)
C10	0.0492 (14)	0.0270 (12)	0.0527 (14)	−0.0007 (10)	0.0169 (11)	0.0021 (10)
C11	0.0432 (14)	0.0457 (15)	0.0740 (19)	−0.0102 (12)	0.0158 (13)	−0.0316 (14)
C12	0.0306 (11)	0.0400 (12)	0.0520 (13)	−0.0038 (10)	0.0056 (10)	−0.0125 (11)
C13	0.0327 (11)	0.0267 (10)	0.0354 (11)	0.0058 (9)	0.0026 (9)	0.0019 (9)
C14	0.0335 (11)	0.0267 (11)	0.0344 (11)	0.0019 (9)	0.0041 (9)	−0.0036 (9)
C15	0.0386 (12)	0.0287 (11)	0.0354 (11)	0.0042 (9)	0.0051 (10)	−0.0009 (9)
C16	0.0325 (11)	0.0284 (11)	0.0397 (12)	0.0027 (9)	0.0027 (9)	−0.0004 (9)
C17	0.0379 (12)	0.0262 (11)	0.0471 (13)	0.0069 (9)	0.0110 (10)	0.0008 (9)
C18	0.0464 (13)	0.0275 (11)	0.0488 (13)	0.0016 (10)	0.0101 (11)	−0.0074 (10)
C19	0.0429 (13)	0.0281 (12)	0.0448 (13)	−0.0030 (10)	0.0102 (10)	−0.0089 (10)
C20	0.0476 (15)	0.0393 (14)	0.0612 (16)	0.0113 (11)	0.0191 (12)	−0.0049 (12)
C21	0.0541 (15)	0.0531 (16)	0.0594 (16)	0.0007 (13)	0.0268 (13)	−0.0073 (13)
C22	0.0497 (14)	0.0460 (15)	0.0488 (14)	−0.0057 (12)	0.0144 (11)	−0.0037 (12)
C23	0.0424 (13)	0.0363 (13)	0.0416 (13)	−0.0011 (10)	0.0068 (10)	−0.0003 (10)
C24	0.0451 (14)	0.0475 (15)	0.084 (2)	−0.0076 (12)	0.0208 (14)	−0.0105 (15)
C25	0.0755 (19)	0.0542 (17)	0.0652 (18)	−0.0055 (15)	0.0418 (16)	−0.0010 (14)
C26	0.0559 (16)	0.0558 (17)	0.0403 (13)	0.0059 (13)	0.0101 (11)	−0.0091 (12)
C27	0.0602 (16)	0.0487 (15)	0.0409 (13)	0.0173 (13)	0.0080 (11)	0.0033 (11)
C28	0.0357 (11)	0.0447 (14)	0.0363 (12)	0.0013 (10)	0.0036 (9)	−0.0010 (10)
C29	0.074 (2)	0.0428 (16)	0.0670 (18)	0.0079 (14)	0.0207 (16)	0.0121 (13)
C30	0.078 (2)	0.068 (2)	0.0618 (18)	−0.0087 (16)	0.0303 (16)	0.0056 (14)
C31	0.0369 (13)	0.0399 (14)	0.0553 (14)	0.0104 (11)	0.0127 (11)	0.0045 (12)
C32	0.0658 (18)	0.0592 (18)	0.0459 (14)	0.0277 (15)	0.0073 (13)	0.0019 (12)
C33	0.0465 (13)	0.0279 (12)	0.0528 (15)	0.0019 (10)	0.0086 (12)	0.0017 (10)
OW	0.0455 (10)	0.0595 (13)	0.0849 (14)	0.0056 (9)	0.0141 (10)	−0.0206 (11)

### Geometric parameters (Å, °)

**Table:**

O1—C1	1.427 (3)	C16—H16A	0.9800
O1—H1A	0.8200	C17—C31	1.512 (3)
C1—C2	1.508 (4)	C17—C18	1.536 (3)
C1—C6	1.526 (3)	C17—C20	1.543 (3)
C1—H1B	0.9800	C18—C19	1.513 (3)
C2—C3	1.518 (3)	C18—H18A	0.9700
C2—H2A	0.9700	C18—H18B	0.9700
C2—H2B	0.9700	C19—H19A	0.9700
O2—C31	1.209 (3)	C19—H19B	0.9700
O3—C31	1.337 (3)	C20—C21	1.509 (4)
O3—C32	1.423 (3)	C20—H20A	0.9700
C3—C4	1.537 (3)	C20—H20B	0.9700
C3—H3A	0.9700	C21—C22	1.517 (4)
C3—H3B	0.9700	C21—H21A	0.9700
C4—C26	1.538 (3)	C21—H21B	0.9700
C4—C5	1.551 (3)	C22—C30	1.533 (4)
C4—C7	1.569 (3)	C22—C23	1.536 (3)
O4—C33	1.201 (3)	C22—H22A	0.9800
C5—C10	1.531 (3)	C23—C29	1.531 (4)
C5—C6	1.563 (3)	C23—H23A	0.9800
C5—H5A	0.9800	C24—H24A	0.9600
O5—C33	1.301 (3)	C24—H24B	0.9600
O5—H5B	0.8500	C24—H24C	0.9600
C6—C25	1.533 (4)	C25—H25A	0.9600
C6—C24	1.536 (4)	C25—H25B	0.9600
C7—C11	1.528 (3)	C25—H25C	0.9600
C7—C15	1.558 (3)	C26—H26A	0.9600
C7—H7A	0.9800	C26—H26B	0.9600
C9—C10	1.521 (3)	C26—H26C	0.9600
C9—C15	1.530 (3)	C27—H27A	0.9600
C9—H9A	0.9700	C27—H27B	0.9600
C9—H9B	0.9700	C27—H27C	0.9600
C10—H10A	0.9700	C28—H28A	0.9600
C10—H10B	0.9700	C28—H28B	0.9600
C11—C12	1.496 (4)	C28—H28C	0.9600
C11—H11A	0.9700	C29—H29A	0.9600
C11—H11B	0.9700	C29—H29B	0.9600
C12—C13	1.314 (3)	C29—H29C	0.9600
C12—H12A	0.9300	C30—H30A	0.9600
C13—C16	1.527 (3)	C30—H30B	0.9600
C13—C14	1.533 (3)	C30—H30C	0.9600
C14—C28	1.548 (3)	C32—C33	1.508 (4)
C14—C19	1.555 (3)	C32—H32A	0.9700
C14—C15	1.580 (3)	C32—H32B	0.9700
C15—C27	1.550 (3)	OW—HWB	0.8500
C16—C17	1.550 (3)	OW—HWA	0.8634
C16—C23	1.553 (3)		
			
C1—O1—H1A	109.5	C20—C17—C16	111.3 (2)
O1—C1—C2	109.6 (2)	C19—C18—C17	112.19 (18)
O1—C1—C6	108.6 (2)	C19—C18—H18A	109.2
C2—C1—C6	114.7 (2)	C17—C18—H18A	109.2
O1—C1—H1B	107.9	C19—C18—H18B	109.2
C2—C1—H1B	107.9	C17—C18—H18B	109.2
C6—C1—H1B	107.9	H18A—C18—H18B	107.9
C1—C2—C3	111.17 (19)	C18—C19—C14	115.02 (19)
C1—C2—H2A	109.4	C18—C19—H19A	108.5
C3—C2—H2A	109.4	C14—C19—H19A	108.5
C1—C2—H2B	109.4	C18—C19—H19B	108.5
C3—C2—H2B	109.4	C14—C19—H19B	108.5
H2A—C2—H2B	108.0	H19A—C19—H19B	107.5
C31—O3—C32	117.5 (2)	C21—C20—C17	112.9 (2)
C2—C3—C4	113.07 (19)	C21—C20—H20A	109.0
C2—C3—H3A	109.0	C17—C20—H20A	109.0
C4—C3—H3A	109.0	C21—C20—H20B	109.0
C2—C3—H3B	109.0	C17—C20—H20B	109.0
C4—C3—H3B	109.0	H20A—C20—H20B	107.8
H3A—C3—H3B	107.8	C20—C21—C22	111.7 (2)
C3—C4—C26	107.61 (19)	C20—C21—H21A	109.3
C3—C4—C5	107.93 (18)	C22—C21—H21A	109.3
C26—C4—C5	113.6 (2)	C20—C21—H21B	109.3
C3—C4—C7	107.62 (18)	C22—C21—H21B	109.3
C26—C4—C7	113.15 (18)	H21A—C21—H21B	107.9
C5—C4—C7	106.63 (17)	C21—C22—C30	110.3 (2)
C10—C5—C4	110.11 (19)	C21—C22—C23	110.4 (2)
C10—C5—C6	114.61 (19)	C30—C22—C23	111.7 (2)
C4—C5—C6	117.54 (18)	C21—C22—H22A	108.1
C10—C5—H5A	104.3	C30—C22—H22A	108.1
C4—C5—H5A	104.3	C23—C22—H22A	108.1
C6—C5—H5A	104.3	C29—C23—C22	110.7 (2)
C33—O5—H5B	119.3	C29—C23—C16	109.6 (2)
C1—C6—C25	112.0 (2)	C22—C23—C16	111.4 (2)
C1—C6—C24	107.2 (2)	C29—C23—H23A	108.3
C25—C6—C24	107.8 (2)	C22—C23—H23A	108.3
C1—C6—C5	107.09 (19)	C16—C23—H23A	108.3
C25—C6—C5	114.0 (2)	C6—C24—H24A	109.5
C24—C6—C5	108.5 (2)	C6—C24—H24B	109.5
C11—C7—C15	110.25 (19)	H24A—C24—H24B	109.5
C11—C7—C4	113.45 (18)	C6—C24—H24C	109.5
C15—C7—C4	117.41 (18)	H24A—C24—H24C	109.5
C11—C7—H7A	104.8	H24B—C24—H24C	109.5
C15—C7—H7A	104.8	C6—C25—H25A	109.5
C4—C7—H7A	104.8	C6—C25—H25B	109.5
C10—C9—C15	114.85 (19)	H25A—C25—H25B	109.5
C10—C9—H9A	108.6	C6—C25—H25C	109.5
C15—C9—H9A	108.6	H25A—C25—H25C	109.5
C10—C9—H9B	108.6	H25B—C25—H25C	109.5
C15—C9—H9B	108.6	C4—C26—H26A	109.5
H9A—C9—H9B	107.5	C4—C26—H26B	109.5
C9—C10—C5	110.30 (19)	H26A—C26—H26B	109.5
C9—C10—H10A	109.6	C4—C26—H26C	109.5
C5—C10—H10A	109.6	H26A—C26—H26C	109.5
C9—C10—H10B	109.6	H26B—C26—H26C	109.5
C5—C10—H10B	109.6	C15—C27—H27A	109.5
H10A—C10—H10B	108.1	C15—C27—H27B	109.5
C12—C11—C7	113.78 (19)	H27A—C27—H27B	109.5
C12—C11—H11A	108.8	C15—C27—H27C	109.5
C7—C11—H11A	108.8	H27A—C27—H27C	109.5
C12—C11—H11B	108.8	H27B—C27—H27C	109.5
C7—C11—H11B	108.8	C14—C28—H28A	109.5
H11A—C11—H11B	107.7	C14—C28—H28B	109.5
C13—C12—C11	126.7 (2)	H28A—C28—H28B	109.5
C13—C12—H12A	116.7	C14—C28—H28C	109.5
C11—C12—H12A	116.7	H28A—C28—H28C	109.5
C12—C13—C16	118.9 (2)	H28B—C28—H28C	109.5
C12—C13—C14	120.3 (2)	C23—C29—H29A	109.5
C16—C13—C14	120.80 (18)	C23—C29—H29B	109.5
C13—C14—C28	107.08 (18)	H29A—C29—H29B	109.5
C13—C14—C19	111.68 (18)	C23—C29—H29C	109.5
C28—C14—C19	106.42 (18)	H29A—C29—H29C	109.5
C13—C14—C15	109.16 (17)	H29B—C29—H29C	109.5
C28—C14—C15	112.80 (17)	C22—C30—H30A	109.5
C19—C14—C15	109.69 (17)	C22—C30—H30B	109.5
C9—C15—C27	108.7 (2)	H30A—C30—H30B	109.5
C9—C15—C7	109.91 (18)	C22—C30—H30C	109.5
C27—C15—C7	110.09 (19)	H30A—C30—H30C	109.5
C9—C15—C14	110.80 (18)	H30B—C30—H30C	109.5
C27—C15—C14	109.52 (18)	O2—C31—O3	122.5 (2)
C7—C15—C14	107.78 (17)	O2—C31—C17	124.6 (3)
C13—C16—C17	110.48 (19)	O3—C31—C17	112.8 (2)
C13—C16—C23	114.31 (18)	O3—C32—C33	111.5 (2)
C17—C16—C23	112.59 (19)	O3—C32—H32A	109.3
C13—C16—H16A	106.3	C33—C32—H32A	109.3
C17—C16—H16A	106.3	O3—C32—H32B	109.3
C23—C16—H16A	106.3	C33—C32—H32B	109.3
C31—C17—C18	109.12 (19)	H32A—C32—H32B	108.0
C31—C17—C20	103.84 (19)	O4—C33—O5	122.0 (2)
C18—C17—C20	111.50 (19)	O4—C33—C32	122.2 (2)
C31—C17—C16	111.69 (19)	O5—C33—C32	115.7 (2)
C18—C17—C16	109.25 (18)	HWB—OW—HWA	84.8
			
O1—C1—C2—C3	−179.9 (2)	C4—C7—C15—C14	−165.94 (16)
C6—C1—C2—C3	−57.5 (3)	C13—C14—C15—C9	−177.45 (18)
C1—C2—C3—C4	57.0 (3)	C28—C14—C15—C9	−58.5 (2)
C2—C3—C4—C26	70.6 (3)	C19—C14—C15—C9	59.9 (2)
C2—C3—C4—C5	−52.4 (2)	C13—C14—C15—C27	62.6 (2)
C2—C3—C4—C7	−167.17 (19)	C28—C14—C15—C27	−178.5 (2)
C3—C4—C5—C10	−174.95 (18)	C19—C14—C15—C27	−60.1 (2)
C26—C4—C5—C10	65.8 (2)	C13—C14—C15—C7	−57.2 (2)
C7—C4—C5—C10	−59.6 (2)	C28—C14—C15—C7	61.7 (2)
C3—C4—C5—C6	51.3 (3)	C19—C14—C15—C7	−179.83 (18)
C26—C4—C5—C6	−67.9 (3)	C12—C13—C16—C17	−139.4 (2)
C7—C4—C5—C6	166.72 (18)	C14—C13—C16—C17	43.4 (3)
O1—C1—C6—C25	49.3 (3)	C12—C13—C16—C23	92.4 (3)
C2—C1—C6—C25	−73.7 (3)	C14—C13—C16—C23	−84.8 (2)
O1—C1—C6—C24	−68.8 (3)	C13—C16—C17—C31	66.4 (2)
C2—C1—C6—C24	168.3 (2)	C23—C16—C17—C31	−164.42 (19)
O1—C1—C6—C5	174.9 (2)	C13—C16—C17—C18	−54.4 (2)
C2—C1—C6—C5	52.0 (3)	C23—C16—C17—C18	74.8 (2)
C10—C5—C6—C1	177.7 (2)	C13—C16—C17—C20	−177.98 (18)
C4—C5—C6—C1	−50.6 (3)	C23—C16—C17—C20	−48.8 (3)
C10—C5—C6—C25	−57.8 (3)	C31—C17—C18—C19	−60.7 (2)
C4—C5—C6—C25	73.9 (3)	C20—C17—C18—C19	−174.8 (2)
C10—C5—C6—C24	62.3 (3)	C16—C17—C18—C19	61.7 (2)
C4—C5—C6—C24	−166.0 (2)	C17—C18—C19—C14	−53.9 (3)
C3—C4—C7—C11	−61.6 (2)	C13—C14—C19—C18	37.8 (3)
C26—C4—C7—C11	57.2 (3)	C28—C14—C19—C18	−78.7 (2)
C5—C4—C7—C11	−177.2 (2)	C15—C14—C19—C18	158.94 (18)
C3—C4—C7—C15	167.87 (19)	C31—C17—C20—C21	171.4 (2)
C26—C4—C7—C15	−73.4 (3)	C18—C17—C20—C21	−71.2 (3)
C5—C4—C7—C15	52.3 (2)	C16—C17—C20—C21	51.1 (3)
C15—C9—C10—C5	−56.6 (3)	C17—C20—C21—C22	−56.5 (3)
C4—C5—C10—C9	63.7 (2)	C20—C21—C22—C30	−177.6 (2)
C6—C5—C10—C9	−161.1 (2)	C20—C21—C22—C23	58.5 (3)
C15—C7—C11—C12	−35.2 (3)	C21—C22—C23—C29	−178.4 (2)
C4—C7—C11—C12	−169.2 (2)	C30—C22—C23—C29	58.4 (3)
C7—C11—C12—C13	3.6 (4)	C21—C22—C23—C16	−56.1 (3)
C11—C12—C13—C16	−177.1 (2)	C30—C22—C23—C16	−179.3 (2)
C11—C12—C13—C14	0.2 (4)	C13—C16—C23—C29	−57.9 (3)
C12—C13—C14—C28	−95.1 (2)	C17—C16—C23—C29	175.0 (2)
C16—C13—C14—C28	82.1 (2)	C13—C16—C23—C22	179.22 (19)
C12—C13—C14—C19	148.8 (2)	C17—C16—C23—C22	52.1 (3)
C16—C13—C14—C19	−34.0 (3)	C32—O3—C31—O2	−1.8 (4)
C12—C13—C14—C15	27.3 (3)	C32—O3—C31—C17	175.0 (2)
C16—C13—C14—C15	−155.50 (18)	C18—C17—C31—O2	−32.5 (3)
C10—C9—C15—C27	−74.9 (2)	C20—C17—C31—O2	86.5 (3)
C10—C9—C15—C7	45.6 (3)	C16—C17—C31—O2	−153.4 (2)
C10—C9—C15—C14	164.66 (18)	C18—C17—C31—O3	150.77 (19)
C11—C7—C15—C9	−177.09 (19)	C20—C17—C31—O3	−90.2 (2)
C4—C7—C15—C9	−45.1 (3)	C16—C17—C31—O3	29.9 (3)
C11—C7—C15—C27	−57.3 (3)	C31—O3—C32—C33	−81.0 (3)
C4—C7—C15—C27	74.7 (2)	O3—C32—C33—O4	10.7 (4)
C11—C7—C15—C14	62.1 (2)	O3—C32—C33—O5	−171.2 (2)

### Hydrogen-bond geometry (Å, °)

**Table:**

*D*—H···*A*	*D*—H	H···*A*	*D*···*A*	*D*—H···*A*
OW—HWB···O2^i^	0.85	2.41	2.839 (3)	112
OW—HWB···O4^i^	0.85	2.58	3.365 (3)	154
O1—H1A···O4^ii^	0.82	1.98	2.758 (3)	159
OW—HWA···O1^iii^	0.86	1.88	2.681 (3)	154
O5—H5B···OW	0.85	1.74	2.575 (3)	165
C12—H12A···OW^i^	0.93	2.57	3.455 (3)	159

Symmetry codes: (i) −*x*, *y*−1/2, −*z*+1; (ii) *x*+1, *y*−1, *z*; (iii) −*x*+1, *y*+1/2, −*z*+1.

## References

[bb1] Allen, F. H., Kennard, O., Watson, D. G., Brammer, L., Orpen, A. G. & Taylor, R. (1987). *J. Chem. Soc. Perkin Trans. 2*, pp. S1–19.

[bb2] Enraf–Nonius (1994). *CAD-4 EXPRESS* Enraf–Nonius, Delft, The Netherlands.

[bb3] Es-saady, D., Simon, A., Ollier, M., Maurizis, J. C., Chulia, A. J. & Delage, C. (1996). *Cancer Lett.* **106**, 193–197. 10.1016/0304-3835(96)04312-1 8844972

[bb4] Harms, K. & Wocadlo, S. (1995). *XCAD4* University of Marburg, Germany.

[bb5] North, A. C. T., Phillips, D. C. & Mathews, F. S. (1968). *Acta Cryst.* A**24**, 351–359.

[bb6] Paton, W. F. & Paul, I. C. (1979). *Cryst. Struct. Commun.* **8**, 207–211.

[bb7] Sheldrick, G. M. (2008). *Acta Cryst.* A**64**, 112–122. 10.1107/S0108767307043930 18156677

[bb8] Simon, A., Delage, C., Saux, M., Chulia, A. J., Najid, A. & Rigaud, M. (1992). *Acta Cryst.* C**48**, 726–728.

[bb9] Stout, G. H. & Stevens, K. L. (1963). *J. Org. Chem.* **28**, 1259–1262.

[bb10] Wen, X.-A., Zhang, P., Liu, J., Zhang, L.-Y., Wu, X.-M., Ni, P.-Z. & Sun, H.-B. (2006). *Bioorg. Med. Chem. Lett.* **16**, 722–726. 10.1016/j.bmcl.2005.10.014 16246555

